# Correlation of Salivary Insulin-Like Growth Factor-I With the Middle Phalanx of the Third Finger (MP3) Skeletal Maturation Stages Across Pubertal Transitions: A Cross-Sectional Study

**DOI:** 10.7759/cureus.113871

**Published:** 2026-08-03

**Authors:** Neharika Awod, Archana Jatania, Manish S Dagdiya, Amit Akolkar, Shakeb Ahemad, Mandar Pathak, Akash Niras, Shruti Tayade

**Affiliations:** 1 Department of Orthodontics and Dentofacial Orthopedics, Saraswati Dhanwantari Dental College and Hospital, Parbhani, IND

**Keywords:** growth assessment, insulin-like growth factor-i, middle phalanx, orthodontics, skeletal maturation

## Abstract

Introduction: Accurate assessment of skeletal maturity is fundamental for planning growth-dependent orthodontic treatments. Although radiographic methods, such as the middle phalanx of the third finger (MP3) skeletal maturation staging, are widely accepted, biological markers have emerged as promising adjuncts for evaluating growth potential. Insulin-like growth factor-I (IGF-I), a key mediator of growth hormone activity, reflects skeletal growth and may provide additional information on pubertal maturation. This study aimed to evaluate salivary IGF-I levels in individuals at different stages of growth and determine their correlation with the MP3 skeletal maturation stages. Materials and methods: A cross-sectional study was conducted on 60 healthy participants aged 7-23 years. Salivary IGF-I concentration was quantified using enzyme-linked immunosorbent assay, while skeletal maturity was assessed using the Hägg and Taranger MP3 method from digital radiographs of the MP3. Data were analyzed using one-way analysis of variance with Tukey's post hoc test, chi-square test, and correlation analysis. Statistical significance was set at p < 0.05. Results: Salivary IGF-I levels differed significantly among the growth groups (p < 0.001). The mean salivary IGF-I concentration increased markedly from the pre-pubertal group (24.99 ± 4.45 ng/mL) to the pubertal group (66.78 ± 7.21 ng/mL). It remained comparable in the post-pubertal group (64.90 ± 3.25 ng/mL). Post hoc analysis demonstrated significantly higher salivary IGF-I levels in the pubertal and post-pubertal groups compared with the pre-pubertal group (both p < 0.001). In contrast, no significant difference was observed between the pubertal and post-pubertal groups (p = 0.322). MP3 skeletal maturation stages showed significant progression across the growth groups (p < 0.001). Overall, salivary IGF-I levels demonstrated a significant positive correlation with MP3 skeletal maturation stage (ρ = 0.611, p < 0.001) and chronological age (r = 0.758, p < 0.001). Conclusions: Salivary IGF-I levels increase significantly during puberty and exhibit a strong positive correlation with MP3 skeletal maturation stages. A combined assessment of salivary IGF-I and MP3 staging may provide a reliable approach for evaluating skeletal maturity and optimizing the timing of growth-modification procedures in orthodontic practice.

## Introduction

Accurate assessment of skeletal maturity is fundamental in orthodontic diagnosis and treatment planning because many dentofacial orthopedic procedures depend on the patient's remaining growth potential [1]. The timing of interventions, such as functional appliance therapy, growth modification, rapid maxillary expansion, and orthognathic treatment, is closely associated with the stage of skeletal development rather than chronological age [2]. Since individuals of the same chronological age often exhibit considerable variation in biological maturation, clinicians require reliable indicators that reflect a patient's true growth status. Consequently, the assessment of skeletal maturity has become an indispensable component of contemporary orthodontic practice [3].

Several methods have been proposed to evaluate skeletal maturation, including chronological age, dental age, secondary sexual characteristics, cervical vertebral maturation (CVM), and hand-wrist radiographs [1-3]. Recent advances have demonstrated the potential of artificial intelligence to improve bone age assessment by providing rapid, standardized, and reproducible skeletal maturity evaluation [4]. The radiographic assessment of the hand and wrist has long been regarded as one of the most reliable approaches because it reflects sequential ossification events associated with the pubertal growth spurt [5]. The middle phalanx of the third finger (MP3) offers a simplified method for skeletal maturity assessment by evaluating characteristic changes in the epiphysis and metaphysis [6]. MP3 staging requires only a small intraoral radiographic exposure and provides a practical alternative for identifying different phases of skeletal growth while minimizing radiation exposure compared with conventional hand-wrist radiographs.

Recent advances in growth biology have highlighted the importance of biochemical markers as potential indicators of skeletal maturation in children. Among these, insulin-like growth factor-I (IGF-I) has received considerable attention because it mediates many of the biological actions of growth hormone and plays a central role in longitudinal bone growth, cartilage proliferation, and skeletal development [7,8]. IGF-I concentrations typically increase during the pubertal growth spurt, reach peak values around the period of maximum growth velocity, and subsequently decline as skeletal maturation is completed. Several studies have demonstrated significant associations between IGF-I levels and radiographic skeletal maturity indicators, suggesting that IGF-I may serve as a valuable biological marker for growth assessment [9].

Although previous studies have evaluated the relationship between IGF-I and CVM or conventional hand-wrist skeletal maturity indicators, limited evidence exists regarding its correlation with MP3 skeletal maturation stages across different growth phases [9]. Establishing this relationship may improve the understanding of biological growth patterns and support the use of biochemical markers as adjuncts to radiographic assessment in orthodontic practices. Furthermore, correlating salivary IGF-I levels with MP3 stages may facilitate a more precise identification of the pubertal growth period, thereby enabling optimal timing of growth-modifying orthodontic treatment. The present study aimed to evaluate salivary IGF-I levels in pre-pubertal, pubertal, and post-pubertal individuals and determine their correlation with MP3 skeletal maturation stages. The objectives were to compare salivary IGF-I levels among the three growth groups, assess skeletal maturation using the Hägg and Taranger [10] MP3 staging method, and investigate the relationship between salivary IGF-I concentrations, MP3 skeletal maturation stages, and chronological age.

## Materials and methods

Study design, setting, duration, and ethical considerations

The present study was a hospital-based cross-sectional analytical study conducted in the Department of Orthodontics and Dentofacial Orthopedics at Saraswati Dhanwantari Dental College and Hospital, Parbhani, India. The study was carried out over a period of 12 months (June 2022 to June 2023). Prior to commencement, the study protocol was reviewed and approved by the Institutional Ethics Committee (approval number: SDDCH/Admin./1537/2022 dated May 20, 2022). All procedures were conducted in accordance with the Declaration of Helsinki (2013 revision). Written informed consent was obtained from all adult participants. For participants younger than 18 years, written informed consent was obtained from their parents or legal guardians, along with assent from the participants wherever applicable.

Eligibility criteria

Healthy individuals aged between seven and 23 years who reported to the outpatient clinic and fulfilled the study criteria were considered eligible. Participants were categorized into three equal groups based on chronological age and growth status: Group I (pre-pubertal; 7-12 years), Group II (pubertal; 13-16 years), and Group III (post-pubertal; 17-23 years). Individuals with good general health, no history of previous orthodontic treatment, and willingness to participate were included. Subjects with systemic diseases, endocrine or metabolic disorders affecting growth, congenital craniofacial anomalies, skeletal dysplasia, growth abnormalities, chronic inflammatory conditions, hepatic or renal disorders, pregnancy, recent trauma or fracture involving the hand, current use of medications known to influence growth hormone or IGF-I metabolism (including corticosteroids or growth hormone therapy), xerostomia, salivary gland disorders, conditions or medications affecting salivary secretion, or inability to provide an adequate saliva sample were excluded from the study.

Sample size calculation

The sample size was estimated prospectively using G*Power software version 3.1.9.7 (Heinrich Heine University, Düsseldorf, Germany) by performing an a priori power analysis for an omnibus F-test (one-way analysis of variance (ANOVA)) to compare the salivary IGF-I levels among the three study groups. An anticipated effect size (Cohen's f = 0.45) was adopted from the study by Sinha et al. [7], which evaluated IGF-I as a skeletal maturity indicator. Assuming a two-sided significance level (α) of 0.05 and a statistical power of 80% (1−β = 0.80), the minimum required sample size was calculated as 60 participants, with 20 participants in each group. This sample size was considered adequate to detect statistically significant differences in salivary IGF-I concentrations and MP3 skeletal maturation stages among the three groups.

Study procedure

Following recruitment, demographic details, including age and sex, were recorded using a standardized case record form. Each participant underwent radiographic assessment of the MP3 and unstimulated whole saliva collection for estimation of salivary IGF-I concentration on the same day to minimize biological variation. Saliva samples were collected under standardized conditions to reduce variations associated with circadian rhythm and recent food intake. A digital radiograph of the MP3 of the left hand was obtained using a digital intraoral radiography (RVG) system (Carestream RVG 5200, Carestream Dental LLC, Atlanta, Georgia, USA). Participants were positioned with the palmar aspect of the left hand placed flat over the digital sensor, and standardized exposure parameters were used for all radiographs. Skeletal maturity was assessed according to the Hägg and Taranger MP3 method, which classifies maturation into MP3-F, MP3-FG, MP3-G, MP3-H, and MP3-I stages based on the morphological relationship between the epiphysis and metaphysis of the middle phalanx [10]. Radiographic interpretation was performed independently by a calibrated orthodontist who was blinded to the serum IGF-I values (Table 1).

**Table 1:** Maturity stages of MP3 according to Hägg and Taranger MP3: middle phalanx of the third finger [10]

Stage	Description
MP3-F	The epiphysis is as wide as the metaphysis.
MP3-FG	The epiphysis is as wide as the metaphysis, and there is a distinct medial and/or lateral border forming a line of demarcation at right angles to the distal border.
MP3-G	The sides of the epiphysis have thickened and cap the metaphysis, forming a sharp edge distally at one or both sides.
MP3-H	Fusion of the epiphysis and metaphysis has begun.
MP3-I	Fusion of the epiphysis and metaphysis is completed.

For biochemical analysis, approximately 3-5 mL of unstimulated whole saliva was collected from each participant between 9:00 and 11:00 AM to minimize the effects of circadian variation. Participants were instructed to refrain from eating, drinking (except water), chewing gum, smoking, tooth brushing, or using mouthwash for at least one hour before sample collection. Before collection, participants rinsed their mouths thoroughly with distilled water and rested for five minutes. Saliva was allowed to accumulate naturally in the floor of the mouth and was collected by the passive drooling method into sterile polypropylene collection tubes without stimulation. The samples were immediately placed on ice and transported to the laboratory, where they were centrifuged at 3000 rpm for 15 minutes at 4°C using a laboratory centrifuge (Remi Elektrotechnik Ltd., Mumbai, Maharashtra, India) to remove cellular debris. The clear supernatant was transferred into sterile cryovials and stored at −80°C in an ultra-low temperature freezer (Thermo Scientific™, Thermo Fisher Scientific Inc., Waltham, Massachusetts, USA) until analysis.

Salivary IGF-I concentration was determined quantitatively using a commercially available human IGF-I enzyme-linked immunosorbent assay (ELISA) kit (FineTest, Wuhan Fine Biotech Co., Ltd., Wuhan, Hubei, China) according to the manufacturer's instructions. All reagents were brought to room temperature before analysis. Standards, controls, and salivary specimens were added to the antibody-coated microplate wells, followed by incubation, washing, enzyme conjugate addition, substrate reaction, and stopping solution according to the kit protocol. Optical density was measured at 450 nm using a microplate reader (Bio-Rad Model 680 Microplate Reader, Bio-Rad Laboratories Inc., Hercules, California, USA). Salivary IGF-I concentrations were calculated from the generated standard calibration curve and expressed as ng/mL. Participants were classified into pre-pubertal, pubertal, and post-pubertal groups according to the predefined study criteria, and salivary IGF-I concentrations were correlated with the corresponding MP3 skeletal maturation stages.

Calibration and reliability

Before the commencement of the study, the principal investigator underwent calibration for MP3 skeletal maturity assessment under the supervision of an experienced orthodontist. Twenty radiographs, which were not included in the final study sample, were independently evaluated twice at a two-week interval to assess intra-examiner reliability. Additionally, a second experienced orthodontist independently evaluated the same radiographs to determine inter-examiner reliability. Reliability was assessed using Cohen's kappa coefficient, with a kappa value greater than 0.80 considered indicative of excellent agreement. During laboratory analysis, all ELISA measurements were performed in duplicate, and the laboratory personnel were blinded to the radiographic findings to minimize measurement bias.

Statistical analysis

Data were analyzed using SPSS Statistics version 25.0 (IBM Corp. Released 2017. IBM SPSS Statistics for Windows, Version 25.0. Armonk, NY). The Shapiro-Wilk test was used to assess the normality of continuous variables, while Levene's test evaluated the homogeneity of variances. Since salivary IGF-I values followed a normal distribution, one-way ANOVA was performed to compare mean salivary IGF-I levels among the three growth groups, followed by Tukey's post hoc test for pairwise comparisons. The association between growth groups and MP3 skeletal maturation stages was evaluated using the chi-square test or Fisher's exact test. Correlations among salivary IGF-I levels, MP3 skeletal maturation stages, and chronological age were assessed using Spearman's rank correlation coefficient and Pearson correlation. A two-tailed p < 0.05 was considered statistically significant.

## Results

Sixty participants were included in the study, with 20 participants each in the pre-pubertal, pubertal, and post-pubertal groups. The mean age differed significantly among the three groups (p < 0.001), whereas the distribution of males and females was comparable, with no statistically significant difference (p = 0.817) (Table 2).

**Table 2:** Age and sex distribution of participants according to growth group (N = 60) Data are presented as mean ± standard deviation or frequency (%), and one-way ANOVA was used to compare age; the chi-square test was used to compare sex distribution. *p < 0.05 was considered statistically significant. χ²: chi-square statistic, SD: standard deviation, ANOVA: analysis of variance

Parameter		Group I - pre-pubertal (n = 20)	Group II - pubertal (n = 20)	Group III - post-pubertal (n = 20)	Test statistics	p-value
Age (years)	Mean age ± SD	8.20 ± 1.44	14.15 ± 1.09	21.75 ± 1.59	F = 480.23	<0.001*
Sex, n (%)	Male	10 (50.0)	9 (45.0)	8 (40.0)	χ² = 0.40	0.817
	Female	10 (50.0)	11 (55.0)	12 (60.0)		

Salivary IGF-I levels varied significantly among the three growth groups (Table 3). The mean salivary IGF-I concentration was the lowest in the pre-pubertal group (24.99 ± 4.45 ng/mL), increased markedly during the pubertal period (66.78 ± 7.21 ng/mL), and remained comparable in the post-pubertal group (64.90 ± 3.25 ng/mL). One-way analysis of variance demonstrated a highly significant difference in salivary IGF-I levels among the groups (F = 399.07, p < 0.001; effect size = 0.93).

**Table 3:** Descriptive statistics of salivary IGF-I level (ng/mL) by growth group Data are presented as mean ± SD, median, minimum, and maximum. One-way ANOVA was used; Eta-squared (η²) was the effect size. *p < 0.05 was considered statistically significant. SD: standard deviation, IGF-I: insulin-like growth factor-I, ANOVA: analysis of variance

Group	N	Mean ± SD	Median	Minimum	Maximum	F-value	p-value	Effect size (η²)
Group I - pre-pubertal	20	24.99 ± 4.45	25.05	16.2	34.5	399.07	<0.001*	0.93
Group II - pubertal	20	66.78 ± 7.21	66.00	54.3	79.4			
Group III - post-pubertal	20	64.90 ± 3.25	65.45	59.2	69.8			

Pairwise post hoc (Tukey) comparisons showed that salivary IGF-I levels were significantly higher in both the pubertal and post-pubertal groups than in the pre-pubertal group (both p < 0.001). In contrast, no statistically significant difference was observed between the pubertal and post-pubertal groups (p = 0.322), indicating that salivary IGF-I levels increased during puberty before reaching a plateau (Table 4).

**Table 4:** Pairwise post hoc comparisons of salivary IGF-I levels between growth groups Tukey's post hoc test was used following one-way ANOVA. *p < 0.05 was considered statistically significant. Group I = pre-pubertal, Group II = pubertal, Group III = post-pubertal. IGF-I: insulin-like growth factor-I, ANOVA: analysis of variance

Comparison	Mean difference	t-statistic	p-value	Effect size (Cohen's d)
Group I vs Group II	-41.79	-21.85	<0.001*	6.91
Group I vs Group III	-39.91	-32.14	<0.001*	10.16
Group II vs Group III	1.88	1.01	0.322	0.32

The distribution of MP3 skeletal maturation stages differed significantly among the study groups (p < 0.001) (Table 5). The pre-pubertal group predominantly exhibited the MP3-F and MP3-FG stages, whereas the pubertal group mainly comprised the MP3-G and MP3-H stages. The post-pubertal group was characterized predominantly by the MP3-I stage, reflecting the completion of skeletal maturation.

**Table 5:** Distribution of MP3 skeletal maturation stages according to growth group Data are presented as frequency (%). Because 40% of the cells had expected frequencies below 5, Fisher’s exact test (Freeman-Halton extension) was applied instead of the chi-square test. *p < 0.05 was considered statistically significant. MP3: middle phalanx of the third finger, df: degrees of freedom

Group	MP3-F	MP3-FG	MP3-G	MP3-H	MP3-I	p-value
Group I - pre-pubertal, n (%)	16 (26.7)	4 (6.7)	0 (0.0)	0 (0.0)	0 (0.0)	<0.001*
Group II - pubertal, n (%)	0 (0.0)	0 (0.0)	7 (11.7)	13 (21.7)	0 (0.0)	
Group III - post-pubertal, n (%)	0 (0.0)	0 (0.0)	0 (0.0)	2 (3.3)	18 (30.0)	

**Figure 1:**
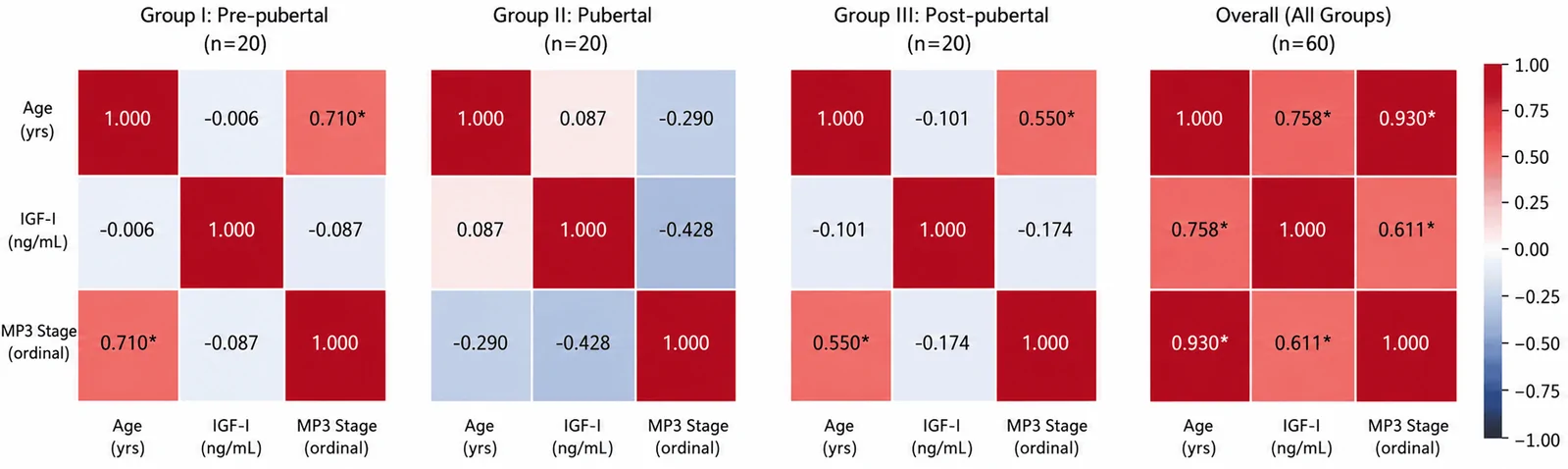
Correlation heat map showing the relationship between chronological age, salivary IGF-I levels, and MP3 skeletal maturation stage in the pre-pubertal, pubertal, post-pubertal, and overall study groups Heat maps illustrate Pearson's correlation between chronological age and salivary IGF-I levels. Separately, Spearman's rank correlation coefficients (ρ) were used to evaluate the association between MP3 skeletal maturation stages and both chronological age and salivary IGF-I levels across pubertal groups. Red indicates positive correlations, blue indicates negative correlations, and color intensity represents the strength of the correlation. Asterisks (*) denote statistically significant correlations (p < 0.05). IGF-I: insulin-like growth factor-I, MP3: middle phalanx of the third finger

Correlation analysis demonstrated a significant positive association between salivary IGF-I levels and the MP3 skeletal maturation stage in the overall sample (Spearman's ρ = 0.611, p < 0.001). Salivary IGF-I levels also showed a strong positive correlation with chronological age (r = 0.758, p < 0.001). In contrast, chronological age demonstrated a very strong positive correlation with the MP3 skeletal maturation stage (ρ = 0.930, p < 0.001) (Table 6, Figure 1). Within-group analyses revealed no statistically significant correlations between salivary IGF-I levels and the MP3 skeletal maturation stage or chronological age in any of the three growth groups. However, chronological age showed a significant positive correlation with the MP3 skeletal maturation stage in the pre-pubertal (ρ = 0.710, p < 0.001) and post-pubertal (ρ = 0.550, p = 0.012) groups. In contrast, no significant association was observed in the pubertal group (ρ = −0.290, p = 0.214). These findings indicate that the overall correlation between salivary IGF-I levels and skeletal maturation primarily reflects differences between growth phases rather than continuous within-stage associations (Table 6, Figure 1).

**Table 6:** Correlation between salivary IGF-I levels, chronological age, and MP3 skeletal maturation stage Spearman's rank correlation test and Pearson's correlation analysis were used. *p < 0.05 was considered statistically significant. IGF-I: insulin-like growth factor-I, MP3: middle phalanx of the third finger, ρ: Spearman's rank correlation coefficient, r: Pearson's correlation coefficient

Group	IGF-I vs MP3 stage (ρ)	p-value	IGF-I vs age (r)	p-value	MP3 stage vs age (ρ)	p-value
Overall	0.611	<0.001*	0.758	<0.001*	0.930	<0.001*
Group I (pre-pubertal)	-0.087	0.716	-0.006	0.981	0.710	<0.001*
Group II (pubertal)	-0.428	0.060	0.087	0.716	-0.290	0.214
Group III (post-pubertal)	-0.174	0.464	-0.101	0.672	0.550	0.012*

## Discussion

Accurate assessment of skeletal maturation is essential for determining the optimal timing of growth modification procedures in orthodontics [1-3]. Although radiographic methods remain the standard for assessing skeletal maturity, biochemical markers, such as IGF-I, have gained increasing attention because they directly reflect the biological processes underlying skeletal growth [7,8]. In the present study, we evaluated salivary IGF-I levels across different growth phases and correlated them with MP3 skeletal maturation stages. The findings demonstrated that salivary IGF-I levels varied significantly among the growth groups, increased markedly during puberty, and showed a significant positive correlation with MP3 skeletal maturation stage and chronological age.

The present study demonstrated significantly lower salivary IGF-I levels in the pre-pubertal group, with a marked increase during the pubertal period, followed by relatively stable concentrations in the post-pubertal group. These findings are consistent with the physiological role of IGF-I as the principal mediator of growth hormone activity during adolescence [8]. Increased secretion of growth hormone during puberty stimulates hepatic IGF-I production, resulting in accelerated chondrocyte proliferation, longitudinal bone growth, and increased skeletal maturation [11,12]. Following the completion of the pubertal growth spurt, growth hormone secretion gradually declines, explaining the stabilization of salivary IGF-I concentrations observed in the post-pubertal group.

These observations are in agreement with those of Masoud et al. [13], who reported that serum IGF-I levels increased significantly during pubertal skeletal maturation stages and reached peak concentrations around the period of maximum growth velocity. Similarly, Gupta et al. [8] demonstrated that serum IGF-I concentrations progressively increased from the pre-pubertal stages, reached their maximum during the MP3-G stage, and subsequently declined toward growth completion. The present findings are consistent with those of Nayak et al. [14], who reported that salivary IGF-I levels peaked during the pubertal growth phase and declined thereafter across CVM stages. Their findings support the role of IGF-I as a useful adjunctive biomarker for assessing skeletal maturation, although it should not be used as a standalone predictor of growth.

The absence of a statistically significant difference in salivary IGF-I levels between the pubertal and post-pubertal groups in the present study suggests that although the greatest increase occurs during puberty, circulating IGF-I remains relatively elevated for a short period before gradually declining toward adult values. This observation is consistent with the meta-analysis by Selva Arockiam et al. [9], who reported that IGF-I shows a strong positive association with skeletal maturity during puberty and subsequently declines after the pubertal growth spurt, supporting its role as a biomarker of skeletal maturation and residual growth potential. The present findings are consistent with those of Almalki et al. [15], who reported low IGF-I levels during the pre-pubertal stage, a marked increase during the pubertal growth spurt, and a subsequent decline toward growth completion, supporting the utility of IGF-I as a biochemical marker of skeletal maturation.

The present study also demonstrated a highly significant association between MP3 skeletal maturation stages and growth phases. Earlier MP3 stages predominated during the pre-pubertal period, whereas advanced stages were increasingly observed during puberty and after growth completion [6]. These findings confirm the validity of the Hägg and Taranger MP3 method as a reliable radiographic indicator of skeletal maturation. Mirabelli et al. [16] reported excellent agreement (88.8%) between the middle phalanx maturation method and CVM, confirming that the MP3 method is a valid alternative for identifying the pubertal growth peak.

An important finding of the present study was the significant positive correlation between salivary IGF-I levels and the MP3 skeletal maturation stage in the pooled sample. This relationship indicates that increasing salivary IGF-I concentrations are accompanied by progressive skeletal maturation. These findings are consistent with those of Carelli et al. [17], who reported a moderate positive correlation between serum IGF-I concentrations and both hand-wrist skeletal maturation indicators and CVM, while observing a strong correlation between the two skeletal maturity indicators. The authors suggested that although serum IGF-I reflects pubertal growth activity, skeletal maturity indicators derived from radiographic assessments remain more reliable for identifying the stages of skeletal development. Similarly, the present study found that the association between IGF-I and skeletal maturation was evident primarily when the entire growth spectrum was considered. In contrast, within-group correlations were weak and non-significant, highlighting that salivary IGF-I is a useful adjunctive biomarker but should not be considered a standalone indicator of skeletal maturity.

Although the overall correlation was statistically significant, within-group analyses revealed weak and non-significant associations between salivary IGF-I levels and the MP3 stage. This finding suggests that salivary IGF-I is most useful for differentiating the broad phases of skeletal maturation rather than detecting minor variations within a single growth stage. Leite et al. [18] further demonstrated that serum IGF-I is a reliable biomarker of pubertal progression, supporting its potential clinical utility in assessing growth and maturation in children. Overall, the findings of the present study strengthen the growing evidence supporting salivary IGF-I as a reliable biological marker of skeletal maturation. When interpreted together with MP3 skeletal maturation staging, salivary IGF-I levels may provide a more comprehensive assessment of growth status and improve the timing of orthodontic growth-modification procedures.

Clinical implications

Salivary IGF-I may serve as a valuable adjunctive biomarker for assessing skeletal maturation in patients undergoing orthodontic treatment. Combined evaluation of salivary IGF-I and MP3 skeletal maturation stages can improve the identification of the pubertal growth spurt, thereby facilitating optimal timing of functional orthopedic treatment, growth modification, and other growth-dependent orthodontic interventions. This combined approach may reduce dependence on chronological age alone and improve individualized treatment planning.

Limitations

The present study had certain limitations. The cross-sectional design did not permit the evaluation of longitudinal changes in salivary IGF-I levels throughout growth. The study was conducted at a single institution with a relatively small sample size, which may limit the generalizability of our findings. Additionally, factors known to influence circulating IGF-I concentrations, including nutritional status, body mass index, physical activity, and sex hormone levels, were not evaluated. Although saliva collection was standardized between 9:00 AM and 11:00 AM to minimize circadian variation, samples were obtained from different participants on different days, and residual day-to-day biological variability cannot be excluded. Furthermore, menstrual-cycle phase was not recorded or standardized among post-menarcheal female participants, which may have contributed to variability in salivary IGF-I concentrations. These factors should be considered when interpreting the findings and should be controlled more rigorously in future longitudinal studies.

Future recommendations

Future multicenter longitudinal studies involving larger and more diverse populations are warranted to validate these findings. Simultaneous evaluation of salivary IGF-I levels with other biochemical markers, such as IGF binding protein-3, alkaline phosphatase, and vitamin D-binding protein, may improve the prediction of skeletal maturity. Further studies integrating biochemical markers with artificial intelligence-assisted radiographic analysis may enhance the precision of growth assessment in orthodontic practice.

## Conclusions

The present study demonstrated that salivary IGF-I levels increased significantly during puberty and showed a strong positive correlation with MP3 skeletal maturation stages. These findings indicate that salivary IGF-I is a reliable biological indicator of skeletal growth and may serve as a useful adjunct to radiographic assessment of skeletal maturity. Combined evaluation of salivary IGF-I and MP3 staging may facilitate more accurate identification of the pubertal growth spurt and improve the timing of growth-dependent orthodontic treatment.
